# Clinical outcomes of low-dose leflunomide for rheumatoid arthritis complicated with Hepatitis B virus carriage and safety observation

**DOI:** 10.12669/pjms.312.6673

**Published:** 2015

**Authors:** Hua Ming-Xu, Meng Chen, Yun Cai, Hui Yan-Jia

**Affiliations:** 1 Ming-Hua Xu, Department of Rheumatology, Affiliated Hospital of Hebei University, Baoding 071000, P.R. China; 2 Meng Chen, Department of Rheumatology, Graduate School of Hebei Medical University, Shijiazhuang 050017, P.R. China. Affiliated Hospital of Hebei University, Baoding 071000, P.R. China; 3 Yun Cai, Department of Internal Medicine-Neurology, Affiliated Hospital of Hebei University, Baoding 071000, P.R. China; 4 Yan-Hui Jia, Department of Rheumatology, Affiliated Hospital of Hebei University, Baoding 071000, P.R. China

**Keywords:** Clinical outcome, Hepatitis B, Rheumatoid arthritis, Leflunomide, Safety

## Abstract

**Objective:**

To study the clinical outcomes of low-dose leflunomide for rheumatoid arthritis (RA) complicated with hepatitis B virus (HBV) carriage and to observe the safety.

**Methods:**

A total of 115 RA patients were divided into three groups according to the state of HBV. They were all given leflunomide to observe the clinical outcomes and whether HBV was activated.

**Results:**

The indices (e.g. activity score) of all patients were significantly better after treatment than those before (P < 0.05), with 89.00% (92/115) of them reaching ACR20. Fourteen cases (12.2%) suffered from abnormal liver functions, and 5 cases who had HBV reactivation originated from the HBV carriage group. Neither the previous HBV infection group nor the infection-free group succumbed to HBV reactivation. The multiple regression model showed that the HBV reactivation risk of RA patients treated by leflunomide was increased by 30% by the basic state of hepatitis B as well as alanine transaminase level and swollen joint count before treatment.

**Conclusion:**

Leflunomide exerted satisfactory therapeutic effects on RA, but liver diseases, liver function, HBV-DNA load and the reactivation risks of carried HBV should be thoroughly checked and cautiously pondered.

## INTRODUCTION

Rheumatoid arthritis (RA), as a systemic autoimmune disease typified by chronic erosive arthritis, is accompanied by synovitis, the resultant destruction of articular cartilage and bone, and eventually joint deformity. Leflunomide (LEF), which is a synthesized disease-modifying antirheumatic drug (DMARD), was the first worldwide approved agent for RA to effectively control disease progression and to hinder bone destruction. However, patients with liver dysfunction should take LEF cautiously due to potentially severe liver injury. Therefore, the effectiveness and safety of LEF for RA patients infected with hepatitis B virus (HBV) still need to be studied. In this prospective study, we evaluated the clinical outcomes of low-dose leflunomide (10 mg/d) for RA complicated with HBV carriage and observed the safety.

## METHODS

### Subjects

RA patients treated in our hospital from January 2006 to June 2013 were selected.

### Inclusion criteria

1) In accordance with the classification criteria (2009 edition) of the American College of Rheumatology (ACR) and the European League Against Rheumatism (EULAR); 2) 18-80 years old; 3) liver function indices (levels of serum alanine transaminase (ALT) and aspartate transaminase (AST)) were examined regularly before and during treatment; 4) serum HBV indices were examined at least twice before and during treatment, including levels of HBV surface antigen (HBsAg) and its antibody (anti-HBs), e antigen (HBeAg) and its antibody (anti-HBe), core antibody (anti-HBc) (detected by ELISA), as well as HBV-DNA (detected by real-time PCR, normal value: <1.0×10^3^ copies/mL).

### Exclusion criteria

(patients under one or more of the following circumstances were excluded): 1) With abnormal laboratory examination results: count of peripheral blood leukocytes< 3.5×10^9^/L or count of neutrophils < 1.5×10^9^/L, AST/ALT≥ twice of the upper limit of normal value, or higher-than-normal serum bilirubin level; 2) complicated with other autoimmune diseases such as systemic lupus erythematosus, and the related symptoms and signs may interfere with the evaluation on drugs; 3) complicated with active hepatitis, hepatitis C, liver cirrhosis, or liver cancer; 4) alcoholism; 5) women planning to have children, pregnant or lactating women; 6) with severe adverse reactions or events induced by LEF before screening, or unrelieved mild-to-moderate adverse reactions.

HBV reactivation was defined as increase of serum HBV-DNA load over 10-fold compared with the baseline level, or transformation of HBsAg/HBeAg from negative to positive. Hepatitis was defined as higher-than-twice of normal ALT/AST upper limit (>80 U/L), accompanied by jaundice or not.^1^

### Grouping and treatment protocols

The patients were divided into the following three groups according to the serum HBV indices before treatment:

1) RA patients carrying HBV, i.e. chronic HBV infection group (positive HBsAg, normal HBV-DNA):
  Low-dose LEF (10 mg/d, Lansen Pharmaceutical Holdings Ltd.) was given for at least 6 months, and nuclear celecoxib (acid) analogues were given to the affordable patients to perform preventive anti-viral therapy.
2) Previous HBV infection group (negative HBsAg and HBV-DNA, positive anti-HBe and/or anti-HBc): This group was administered with 10 mg/d LEF for at least 6 months.
3) Infection-free group (positive anti-HBs, with all the other serum HBV indices being negative): This group was administered with 10 mg/d LEF for at least 6 months.

For all the three groups, the patients obviously suffering from pain were additionally administered with non-steroidal anti-inflammatory drugs. Clinical outcomes were evaluated every month, and liver functions, HBsAg index and HBV-DNA level were also detected.

### Standards for evaluating clinical outcomes

The classification criteria of ACR, i.e. ACR20 and DAS28, were employed to evaluate the clinical outcomes. Definition of ACR20: Swollen joint count (SJC28) and tender joint count (TJC28) were improved by 20%, and at least three out of the five items below were improved: 1) Pain score (rest pain) of subjects; 2) overall disease score of subjects; 3) overall disease score evaluated by the authors; 4) health assessment questionnaire (HAQ); 5) acute-phase reactants (ESR and CRP).

### Indices for safety observation

Indices for observing liver safety: Liver function indices (levels of AST, ALT, gamma glutamyl transpeptidase (GGT) and bilirubin) were detected every month before and after treatment. The HBV-DNA levels of HBV carriers were rechecked, and the HBsAg levels of previous infection group or infection-free group were rechecked. Other safety indices: Routine blood test and renal function examination were conducted in the 1st, 3rd and 6th months after treatment. Electrocardiogram and chest X-ray were recorded before and 6 months after treatment.

### Statistical analysis

All data were analyzed by SPSS 19.0. The categorical data were expressed as mean ± standard deviation or medians (interquartile range). Categorical variables among groups were compared by χ^2^ test or Fisher’s exact test. Intra-group comparisons before and after treatment were performed with paired t-test. Risk factors of HBV reactivation were screened by using multiple regression analysis with stepwise regression method, with the significance level of 0.05.

## RESULTS

### Baseline clinical data

A total of 115 patients completed the follow up, including 33 males and 82 females aged 19-78 years old (average: 53 ± 12). They were followed up for 1-88 months, with the median time of 31 months. There were 17 cases of chronic HBV infection (2 cases of initially being complicated with positive HBV-DNA (6.17×10^7^ copies/mL. 3.29×10^5^ copies/mL)), 36 cases of previous HBV infection, and 62 cases of infection-free. The gender, age, disease course and follow-up time of the three groups were similar (P > 0.05) (Table-I).

**Table-I:** Baseline clinical data of different HBV infection groups (x ± s).

Group	Case No.	Female		Age	Disease course	Follow-up time
		Case No.	%	(year)	(month)	(month)
Chronic HBV infection	17	13	76	47±13	13 (2-360)	40 (2-85)
Previous HBV infection	36	26	72	53±13	37 (1-360)	35 (1-86)
HBV infection-free	62	43	69	54±14	49 (1-360)	30 (1-88)

### Clinical outcome evaluation

After 24 weeks of treatment, 79.13% (91/115) of the patients reached ACR20, including 62.5% (10/16) of the chronic HBV infection group, 72.22% (26/36) of the previous HBV infection group, and 87.30% (55/63) of the infection-free group. After LEF treatment, TJC28, SJC28, CRP level and disease activity score (DAS28)-C-reactive protein (CRP) values were significantly improved compared with those before (P < 0.05). The DAS28-CRP values of the three groups were similar after treatment (P > 0.05) (Table-II).

**Table-II:** Clinical outcomes before and after LEF treatment (x± s).

Group	Before				After			
	TJC28	SJC28	CRP (mg/L)	DAS28-CRP	TJC28	SJC28	CRP (mg/L)	DAS28-CRP
Chronic HBV infection	10.1±7.7	7.9±7.7	37.8±50.8	4.8±1.7	6.3±5.7	5.4±5.9	8.4±12.6	3.8±1.5
Previous HBV infection	10.7±8.7	6.8±6.3	46.1±44.9	5.1±1.3	3.9±3.8	3.4±2.8	2.8±1.8	3.2±1.1
Infection-free	12.1±8.7	9.7±7.5	41.4±38.3	5.4±1.3	4.5±3.8	3.8±2.8	2.4±1.5	3.3±9.4

P < 0.05.

### Liver functions

The AST and ALT levels of all groups were elevated after treatment, and 14 cases suffered from liver dysfunction (significant increase of AST or ALT level twice of the normal upper limit), including 5 cases from the chronic HBV infection group, 5 cases from the previous HBV infection group and 4 cases from the infection-free group. Five cases of the chronic HBV infection group were subjected to reactivation (31.3%), but the other two groups were not. Other indices of the same group had statistically significant differences before and after treatment (P < 0.05) (Table-III).

**Table-III:** Liver functions before and after treatment (x ± s).

Group	Before		After		HBV reactivation	
	AST (U/L)	ALT (U/L)	AST (U/L)	ALT (U/L)	Case No.	%
Chronic HBV infection	38±56	49±108	128±214	152±253	5	31.3
Previous HBV infection	21±10	19±22	57±64	64±77	0	0
Infection-free	23±18	21±19	46±42	47±55	0	0

P < 0.05.

### Reactivation of chronic HBV infection group

After treatment, HBV-DNA levels of 8 cases (50%) in the chronic HBV infection group rose, and 5 cases (31.3%) had HBV reactivation. Of the 5 reactivated cases, there were two cases of jaundice-free hepatitis (40%) (Case 6 was relieved by using other RA drugs plus antiviral therapy with lamivudine; Case 5 stopped using LEF several times and did not receive further treatment), two cases of jaundice-accompanied hepatitis (40%) (Case 7 was diagnosed by abdominal ultrasound as fatty liver disease, and finally died of liver failure after stopping using LEF, antiviral therapy with lamivudine and liver case therapy; Case 8 was administered with sulfasalazine pyrimidine instead of LEF, and also given lamivudine and liver care therapy for four months until the transaminase levels returned to normal and HBV-DNA became negative), as well as one case of asymptomatic HBV reactivation (20%) (Case 4 was administered with other RA drugs plus antiviral therapy with lamivudine) (Table-IV).

**Table-IV:** Outcomes of patients with HBV-DNA increases after treatment.

Disease course	Age(year)	Gender	HBsAg	Baseline HBV-DNA (copies/mL)	After			
					HBV-DNA (copies/mL)	AST (U/L)	ALT (U/L)	Liver state
1	42	Female	Positive	<1.0×103	1.93×103	29	39	Asymptomatic HBV-DNA increase
2	39	Female	Positive	<1.0×103	7.88×103	29	37	Asymptomatic HBV-DNA increase
3	55	Female	Positive	<1.0×103	4.0×103	116	124	HBV-DNA increase, jaundice-free hepatitis
4	53	Male	Positive	<1.0×103	7.66×105	29	21	Asymptomatic HBV-DNA reactivation
5	29	Female	Positive	7.14×103	9.92×105	48	104	HBV reactivation, jaundice-free hepatitis
6	53	Female	Positive	<1.0×103	9.66×105	327	741	HBV reactivation, jaundice-free hepatitis
7	58	Female	Positive	<1.0×103	1.04×105	327	202	HBV reactivation, jaundice-accompanied hepatitis
8	17	Female	Positive	3.29×105	1.79×108	834	826	HBV reactivation, jaundice-accompanied hepatitis

### Screening of risk factors by multiple regression analysis

Multiple regression analysis showed that the HBV reactivation risk of RA patients treated by LEF was increased by 30% (adjusted coefficient of determination: 0.290) by the basic state of hepatitis B as well as ALT level and SJC28 before treatment (F = 16.559, P < 0.001). Besides, the standardized partial regression coefficients (0.379, 0.351 and 0.189 for the three factors respectively) suggested that the former two factors were more determinative. On the other hand, age, gender, disease course, TJC28 and DAS28-CRP did not exert significant effects.

## DISCUSSION

LEF can remarkably decrease the aggregation of inflammatory cells at joints^2^ and effectively control disease progression^3^ by inhibiting dihydro-lactate dehydrogenase and tyrosine kinase to reduce pyrimidine formation, by decreasing the number of activated T lymphocytes, and by dramatically suppressing the catalytic capacity of neutrophils. Moreover, LEF is able to obviously improve the quality of life of RA patients, with comparable effects to methotrexate or even better effects.^4^-^6^ In this study, after 24 h of LEF treatment (10 mg/d), TJC28, SJC28, and DAS28-CRP values of 115 RA patients were significantly improved. In the last, 80.00% (92/115) of the patients reached ACR20.

However, LEF leads to drug-induced liver injury or even acute drug-induced hepatitis, probably by inhibiting the activity of CYP2C9 enzyme that catalyzes the metabolisms of many endogenous and exogenous substances in human body.^7^ In addition, the AST and ALT levels of all patients were raised after LEF treatment, of which 14 cases underwent increases twice of the normal limit, including one case of chronic HBV infection complicated with alcoholism who was diagnoses as fatty liver disease, and 5 cases with HBV-DNA increases that were related with hepatitis B activity. In the other two groups, 9 cases undergoing elevated liver enzyme levels and negative HBV-DNA began to show normal transaminase levels after LEF was no longer given. Regardless, LEF-induced liver injury is transient and can be recovered after decreasing its dose or stopping using it.^8^ LEF should not be given any longer if ALT level exceeds three times of the normal values (>120 U/L), and cholestyramine or activated carbon treatment is highly recommended as well. Once ALT level recovers to normal, LEF can be used again safely in most cases under enhanced liver care and follow up. This study suggested that iatrogenic liver disease may result in severe liver injury and even endanger human life, so organic liver disease should be excluded and liver function indices should be timely monitored before and after treatment.

In the chronic HBV infection group, 5 cases suffered from HBV reactivation, of which 4 cases were administered with other DMARDs, lamivudine and liver care until HBV-DNA turned negative, with the remaining recovering 12, 16 and 19 months later by using the original treatment protocol. Furthermore, 31.3% of the HBV carriers succumbed to reactivation, which were successfully controlled and alleviated by close monitoring and antiviral therapy.

Multiple regression analysis showed that the HBV reactivation risk of RA patients treated by LEF was increased by 30% by the basic state of hepatitis B as well as ALT level and SJC28 before treatment, and the first factor had the most significant influence.

In summary, LEF exerted satisfactory therapeutic effects on RA, but liver diseases, liver function, HBV-DNA load and the reactivation risks of carried HBV should be thoroughly examined and cautiously handled.

## References

[1] Hoofnagle JH (2009). Reactivation of hepatitis B. Hepatology.

[2] Kraan MC, de Koster BM, Elferink JG, Post WJ, Breedveld FC, Tak PP (2000). Inhibition of neutrophil migration soon after initiation of treatment with leflunomide or methotrexate in patients with rheumatoid arthritis: findings in a prospective, randomized, double-blind clinical trial in fifteen patients. Arthritis Rheum.

[3] Kalden JR, Scott DL, Smolen JS, Schattenkirchner M, Rozman B, Williams BD (2001). Improved functional ability in patients with rheumatoid arthritis-longterm treatment with leflunomide versus sulfasalazine. European Leflunomide Study Croup. J Rheumatol.

[4] Golicki D, Niewada M, Lis J, Pol K, Hermanowski T, Tłustochowicz M (2012). Leflunomide in monotherapy of rheumatoid arthritis: meta-analysis of randomised trials. Pol Arch Med Wewn.

[5] Wolfe F, Michaud K, Stephenson B, Doyle J (2003). Toward a definition and method of assessment of treatment failure and treatment effectiveness: the case of leflunomide versus methotrexate. J Rheumatol.

[6] Sakellariou GT, Sayegh FE, Kapetanos GA, Berberidis C (2012). Efficacy of leflunomide addition in relation to prognostic factors for patients with active early rheumatoid arthritis failing to methotrexate in daily practice. Clin Rheumatol.

[7] Kremer JM, Genovese MC, Cannon GW, Caldwell JR, Cush JJ, Furst DE (2002). Concomitant leflunomide therapy in patients with active rheumatoid arthritis despite stable doses of methotrexate. A randomized double-blind, placebo-controlled trial. Arzneimittelforschung.

[8] Gupta R, Bhatia J, Gupta SK (2011). Risk of hepatotoxicity with add-on leflunomide in rheumatoid arthritis patients. Arzneimittelforschung.

